# The Evolutionary Relationship between Alternative Splicing and Gene Duplication

**DOI:** 10.3389/fgene.2017.00014

**Published:** 2017-02-14

**Authors:** Luis P. Iñiguez, Georgina Hernández

**Affiliations:** Programa de Genómica Funcional de Eucariotes, Centro de Ciencias Genómicas, Universidad Nacional Autónoma de MéxicoCuernavaca, México

**Keywords:** alternative splicing, evolution, gene duplication, polyploidy, mRNA isoforms

## Abstract

The protein diversity that exists today has resulted from various evolutionary processes. It is well known that gene duplication (GD) along with the accumulation of mutations are responsible, among other factors, for an increase in the number of different proteins. The gene structure in eukaryotes requires the removal of non-coding sequences, introns, to produce mature mRNAs. This process, known as *cis*-splicing, referred to here as splicing, is regulated by several factors which can lead to numerous splicing arrangements, commonly designated as alternative splicing (AS). AS, producing several transcripts isoforms form a single gene, also increases the protein diversity. However, the evolution and manner for increasing protein variation differs between AS and GD. An important question is how are patterns of AS affected after a GD event. Here, we review the current knowledge of AS and GD, focusing on their evolutionary relationship. These two processes are now considered the main contributors to the increasing protein diversity and therefore their relationship is a relevant, yet understudied, area of evolutionary study.

## Introduction

Organismal protein diversity has increased through evolution. This diversity has allowed organisms to adapt to different environments, to develop and to differentiate specialized tissues. GD, along with mutations, has been an important process to increase the protein diversity. The genome sequence of a variety of organisms has allowed the identification of paralogous genes produced by GD. Nevertheless, the vast amount of proteins cannot be solely explained by this process. Before the human genome was sequenced it was widely believed that the human genome would encode around 100,000 genes based on an estimate of the number of different proteins existing in human cells (Fields et al., 1994). Surprisingly only ∼21,000 coding genes were annotated in the genome sequence (Auton et al., 2015). Almost all protein coding genes from eukaryotes contain non-coding sequences, known as introns, flanked by coding sequences, or exons. During transcription introns need to be removed in order to form a mature mRNA, this process is called splicing. Gilbert (1978) proposed that alterations on the splicing of a gene could form multiple isoforms from a single gene. AS is the process through which a single gene can produce different mRNA isoforms and those in turn, if translated, could lead to multiple proteins. The AS process could explain the discrepancy between the estimates of number of genes and number of proteins.

## Gene Duplication

The vast number of genes in eukaryotic organisms is in large part due to GD. Several processes can occur in the cell that can lead to the duplication of genes. There are two GD classifications; SSD and WGD. SSD duplicates one or several genes while WGD increases significantly the offspring’s gene count compared to the parent. SSDs events may result from unequal cross-over (DNA-dependent) or retrotransposition (RNA-dependent). Both processes give rise to different gene structures. Retrotransposed genes become single exon genes while DNA-dependent duplications inherit gene structure and regulatory sequences (reviewed by Van de Peer et al., 2009). A WGD is caused by autopolyploidy or allopolyploidy (Van de Peer et al., 2009). It has been hypothesized that WGDs provide organisms with certain defenses against extinction, because individuals can accumulate mutations in duplicated genes that may enhance their adaptation to stress or environmental conditions (Innan and Kondrashov, 2010).

Four models have been proposed to describe the evolution of duplicated, or paralogous genes. The model of neofunctionalization establish that one copy of the gene retains the ancestral function while the function of the other diverges into a new one. Subfunctionalization, or duplication-divergence-complementation propose that the ancestral gene function is partitioned between paralogs. Subfunctionalization was tested by Kito et al. (2016) in several yeast species, some of which contained several SSD. Using proteomics, they found that in species that experienced SSD, the sum of paralogous proteins was similar to the amount of the non-duplicated homologous proteins in other yeast species. Another evolutionary model is that one paralog retains the ancestral function while the other paralog devolves into a pseudogene (Panchy et al., 2016). Finally, the function of both paralogs can remain similar if an increased production of the protein is advantageous or if a dosage balance occurs in conjunction with other gene products (Innan and Kondrashov, 2010; Magadum et al., 2013). Wang et al. (2011, 2012) studied the spatiotemporal expression profiles of duplicated genes to identify the different evolutionary models based on the gene expression profiles of paralogous genes. They concluded that the divergence in expression depends on the process of duplication. To complement these studies, Lan and Pritchard (2016) analyzed SSD and found that the genomic distance, the type of duplication and the time since duplication all influence the fate of paralogous genes. These evolutionary models have been studied and reviewed by Cañestro et al. (2013) where examples of each model are described.

## Alternative Splicing

**FIGURE 1:**
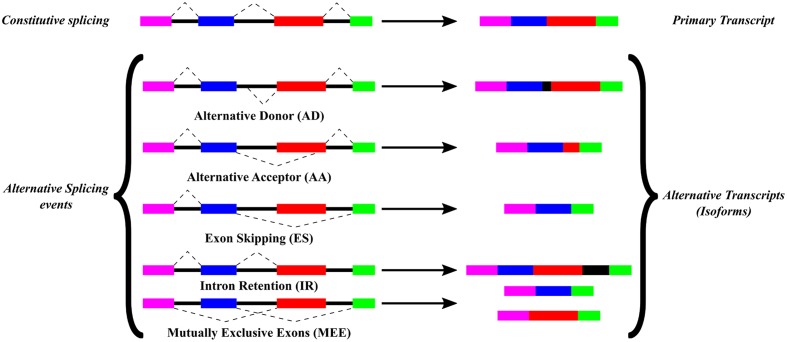
**Alternative splicing events**. Types of AS events, previously described and commented in this work, are based on a comparison between the constitutive splicing and the alternative splicing events for a certain gene. Color boxes represent exons while black lines represent introns from a gene. Splicing sites are depicted by connections with dashed lines.

Alternative splicing is a post-transcriptional process that occurs in different stresses, developmental conditions and in different cell types (reviewed by Wang et al., 2008; Staiger and Brown, 2013; Li et al., 2016). AS affects the localization of the mature mRNA and its translation efficiency (Zhiguo et al., 2013). It can also produce alternative stop codons and can regulate protein expression by non-sense-mediated decay (Kalyna et al., 2012). AS may result in different protein isoforms derived from a single gene and these isoforms alter their cellular localization or function with respect to the primary transcript (Remy et al., 2013). AS can also influence in protein–protein interactions. These interactions have been associated with intrinsically disordered protein domains, which are more susceptible to AS (Niklas et al., 2015; Yang et al., 2016). Five main AS events lead to the production of different isoform transcripts as shown in **Figure 1**: ES, where a complete exon is absent from the primary transcript; AA, where the 3′ end of an intron changes; AD, where the 5′ splice site of the intron is different; IR, where a reported intron is not spliced and is part of the mature mRNA and MEE, where one of two exons is retained in a given isoform but not both exons. These AS events vary in their frequency among different eukaryotic organisms. In animals, ES is the most common AS event, which represents around 50% of all AS events, while in plants IR is the most frequent AS event (reviewed Vélez-Bermúdez and Schmidt, 2014; Zhang et al., 2015).

Nematodes and humans show high variation in cell types and both genomes code for a similar number of genes. However, 98% of human multiple-exon genes exhibit AS (Pan et al., 2008) while AS is present in only 25% of nematode genes (Ramani et al., 2011). Chen et al. (2014) analyzed several organisms which vary in their number of different cell types –referred here as organism complexity- and found a strong positive correlation between the number of cell types and the number of AS events. Organisms with more complexity tend to have higher AS. In addition to AS, it has been found that non-coding RNA’s have a correlation with organismal complexity (Liu et al., 2013). There is evidence that proteome size, structural disorder of proteins, protein–protein interactions and AS are all part of a fine tuning of a complex network to ensure organism complexity (Schad et al., 2011; Dunker et al., 2015).

## The Evolution of as is Closely Linked to GD Events

**FIGURE 2:**
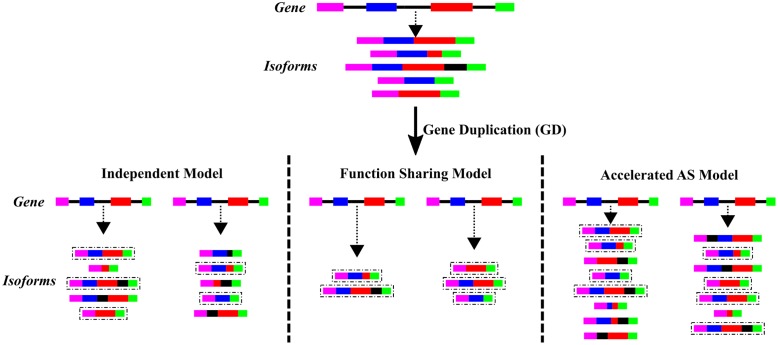
**Evolutionary models of AS after GD**. The three different proposed models of evolution of AS after GD are depicted. Color boxes represent exons while black lines represent introns from a gene. The upper part represents a single-copy gene, before GD, that give rise to the different mRNA isoforms (connected by a dash-line arrow) due to AS. The lower part represents the same gene after GD and each of the possible evolutionary models of AS. The isoforms highlighted in a dashed line box represent those present before GD.

Transcript isoforms resulting from AS events can be viewed as having “internal-paralogs” in the same gene (Kopelman et al., 2005). These “internal-paralogs” may have different functions, similar to the neofunctionalization model of gene evolution. For these reasons the comprehension and analysis of the relationship between AS and GD is an interesting topic in the evolutionary field. It has been observed that mutating a single intronic nucleotide can provoke changes in gene splicing patters (Hsiao et al., 2016), which would facilitate a fast evolution on patterns of AS in paralogus genes. Reddy et al. (2013) reviewed three different models to explain this relationship, these models are summarized in **Figure 2**. The independent model establishes the lack of relationship between GD and AS and therefore the number of isoforms in paralogs vs. non-duplicated genes would be similar. The functional sharing model illustrates the subfunctionalization of the paralogous genes, where one paralog would adopt certain number of ancestral AS events and the other paralog adopts the rest of the ancient isoforms. Therefore, in the functional sharing model the number of AS events per gene decreases in comparison with the non-duplicated genes. The last model is the accelerated AS model, where an increase in the number of AS events per gene results from a relaxed selection pressure for each paralogous gene.

The most common AS event analyzed in paralogous genes is MEE. This AS event accounts for the particularity where given the possibility of two exons, one is maintained in one duplicated gene and lost in the other, and vice versa in the paralog. The first example found to have this pattern was the microphthalmia-associated transcription factor in *Danio rerio* (Altschmied et al., 2002). This is a single copy gene with and at least two mRNA isoforms in mammals. The isoforms from this gene vary in the 3′-end of the mature mRNA and are expressed in different tissues. Altschmied et al. (2002) analyzed this gene in zebrafish, a species from the teleost which have presented several GD events in their evolutionary history. They found two paralogs, one that had one exon while the paralog had the other exon. Both paralogs were expressed in different tissues, thus confirming that GD had replaced AS. Several reports have confirmed the model of function sharing for MEE events in a few genes (Wei-Ping et al., 2003; Pacheco et al., 2004; Cusack and Wolfe, 2007; Hultman et al., 2007; Marshall et al., 2013). To generalize this model, Abascal et al. (2015) analyzed ∼90 human genes exhibiting MEE events. They identified the duplicated orthologous genes in five different fish species, including zebrafish. While several cases were identified that fit the function sharing model, one paralog containing one exon, while the other paralog contained the other exon, not all orthologous genes fit the function sharing model. In this report they also found duplicated genes with the same MEE event observed in humans. This report suggested that a single model could not be generalized, instead each gene possesses a unique AS evolutionary model.

The first attempt to generalize an evolutionary model for AS after GD was proposed by Kopelman et al. (2005). They divided the human genes in families depending on the number of paralogs and determined the proportion of genes affected by AS. They reported that larger gene families tend to have fewer genes affected by AS in comparison with single-copy genes (singletons). They also searched for homologous genes in mouse and classified them into duplicated and non-duplicated genes. They found that duplicated genes were less affected by AS in both organisms. These results were further confirmed by Su et al. (2006), who proposed the function sharing model as the main evolutionary model of AS after GD. In this study they report that AS events are lost in recent gene duplicates but novel AS events are gained in ancient paralogs. They also investigated the symmetry of AS in paralogs, where the number of AS events in the two paralogs are equal. They argued that 16 to 34% of the paralogous genes analyzed exhibit patterns of asymmetric AS. Jin et al. (2008) analyzed the proportion of genes affected by AS and the average number of AS events per family. They found that larger families have a smaller proportion of genes affected by AS and that the number of AS events per gene is lower in larger gene families. Another important aspect to consider in the relationship between GD and AS is the expression patterns of the paralogous genes and their isoforms. Talavera et al. (2007) studied the correlation of expression of paralogs with and without AS events in a set of tissues. They found that duplicated genes without AS have more similar expression patterns compared to the expression pattern between duplicated genes with AS. This result suggests a relationship between gene expression patterns and AS in duplicated genes. Nevertheless, it is not clear if AS controls gene expression or vice versa.

Hughes and Friedman (2008) analysis of the AS and GD relationship in *Caenorhabditis elegans*, confirmed findings from previous analyses in mouse and human indicating that larger gene families had fewer AS events. However, these authors focused their work on an interaction network that consisted of ∼900 genes; they found a negative correlation between AS events and the connectivity in the network. This means that a gene with multiple connections to other genes, also known as a hub, had fewer AS events than genes connected to one or two genes. Further, they argued that duplication of hub genes was an uncommon phenomenon. Therefore AS, interpreted as an internal paralog, was unlikely to occur in hub genes.

Plants are organisms with a tendency to have undergone WGDs throughout their evolutionary history; and are therefore particularly important in the study of AS and GD. Lin et al. (2008) studied families of paralogous genes in *Arabidopsis thaliana* and *Oryza sativa*. They analyzed several factors and characteristics of singletons vs. duplicated genes and found that singletons were less affected by AS than paralogs in both plant species; which contrasts with findings from animal studies. In agreement with these results, Roux and Robinson-Rechavi (2011) found that paralogous families containing exactly two members had more AS events and a higher proportion of these genes are affected by AS than the rest of the other gene families in humans. This is also in agreement with previous reports indicating that larger families have fewer AS events and are less affected than singletons genes. They classified the duplicated genes according to their time of appearance in the evolutionary history, observing a positive correlation between the number of AS events and the time since duplication indicating that duplicated genes acquire AS events over time. They also observed a negative correlation between selective pressure and AS, meaning that paralogous genes under strong positive selection tend to have fewer AS events than paralogs under weak selection. They also searched for orthologous genes and AS events in mouse and found that genes duplicated in human but not in mouse had fewer AS events than genes that have not undergone duplication. They argue that the model best explains AS after GD and, based on the comparison with mouse, genes with fewer AS events tend to duplicate more frequently. Chen et al. (2011) analyzed duplicated genes in human and mouse and observed, based on protein similarity, that the time of duplication is positively correlated with AS event acquisition. This accounts for why ancient paralogs tend to have more AS than recently duplicated genes, which is consistent with the findings described above for plants.

The two contrastingly AS evolutionary models, function sharing and independent, were discussed more recently by Su and Gu (2012). They argued against the acquisition of AS through time and a predisposition of duplication based on their AS events mentioned by Roux and Robinson-Rechavi (2011). These authors also argued the AS evolution after GD is most influenced by the different types of GD. Thus, GD arising from SSD tend to accumulate more mutations than WGD. Such mutations could be a replacement of AS events and therefore AS events are lost quickly. Tack et al. (2014) studied different WGD and tandem duplications in *A. thaliana* looking for the qualitative and quantitative conservation of AS events in paralogs. Such conservation was higher in paralogous genes resulting from tandem duplications than from WGD-resulting paralogs. They found that IR, the most frequent AS event in plants, was also the event with highest conservation in *A. thaliana.* These results suggest that the fate of AS events after GD depends on the type of AS event and the type of GD.

Lambert et al. (2014) analyzed paralogous and singleton genes from three important gene ontology categories in zebrafish to better understand the relationship between AS and GD. The authors investigated the conservation of exon structure in paralogous genes by cataloging genes into paralogs with the same exon structure and paralogs with different exon structures. They found that paralogs with a change in its exon structure tend to have fewer AS events than genes with the same exon structure. They also looked for orthologous genes and their isoforms in human. They found that the percentage of human genes affected by AS and the number of AS events was less in homologous genes with altered exon structure compared to genes with the same exon structure. For this work authors pooled together retrogenes with DNA-dependent SSD which could influence the conclusions. These results refute the hypothesis that genes lacking AS are predisposed to GD events (Roux and Robinson-Rechavi, 2011). Rather, there is the possibility that exon structure of paralogs is predisposed to change if the ancient gene encoded only a low number of AS events. There was no evidence of AS differences between duplicated genes with the same exon structure and singletons. These findings were confirmed by Lambert et al. (2015), where only DNA-dependent SSD were analyzed. This study compared three gene sets from human, mouse and zebrafish: duplicates with same exon structure, duplicates with different exon structure and singletons. They found that AS was less frequent in paralogous genes with different exon structure and these genes exhibited tissue-specific expression. They concluded that paralogs with altered exon structure are subfunctionalized because the expression of the two paralogs occurs in different tissues.

The relationship between GD and AS is far from being understood. Several models of this relationship have been proposed and examples of each have been demonstrated. The analysis of these processes is complex and therefore a generalization of an evolutionary model is a difficult task. The development and utilization of new technologies has allowed the identification of gene isoforms expressed in a tissue-specific or even a cell-specific manner in more model organisms (Conesa et al., 2016). More studies in this field need to be performed to more fully understand this relationship.

## Perspectives

The correct identification and classification of AS and GD is fundamental to improve the understanding of the evolution of both processes. AS events must be classified in terms of how they modify the primary transcript and the expression of unique isoforms in specific conditions, tissues and developmental stages. Future studies should also consider the percentage of affected genes and that the frequency of different AS event types varies between plants and animals. The classification of AS events should also be complemented by identifying and classifying GD events. The time elapsed after a GD event is an important factor in paralogous gene AS. In addition, this could be complemented with the mutation rates for each gene. These could give insights of the evolution of the paralogous genes. Besides classifying GD events as either SSD or WGD, researchers must also determine the way they were produced and the time since the duplication is necessary. For this classification, the comparison between species is important. There are a variety of model species, each uniquely qualifies as a study organism, for the different GD processes. Plants and teleost species in the animal kingdom, for example, are good models for the classification of WGD and SSD, respectively. Several organisms and a variety of tissues and conditions need to be analyzed with a characterization and classification of type of AS and GD in order to identify an evolutionary model of AS events after GD. These analyses could be complemented with other genome-wide analysis including isoforms quantification and proteomic studies (Payne, 2015). A single evolutionary model of AS after GD may not be solely responsible for AS events, instead a combination of multiple models is more likely. Identifying the mechanisms governing which models are utilized in specific genes will improve our understanding of the evolutionary relationship between GD and AS.

## Author Contributions

All authors listed, have made substantial, direct and intellectual contribution to the work, and approved it for publication.

## Conflict of Interest Statement

The authors declare that the research was conducted in the absence of any commercial or financial relationships that could be construed as a potential conflict of interest.

## Abbreviations

AA: alternative acceptor
AD: alternative donor
AS: alternative splicing
ES: exon skipping
GD: gene duplication
IR: intron retention
MEE: mutually exclusive exon
SSD: short segmental duplication
WGD: whole genome duplication
